# A Comparison Between the Effectiveness of Ketamine Bolus and Intradermal Lidocaine in Reducing Acute Postoperative Pain

**DOI:** 10.7759/cureus.26563

**Published:** 2022-07-04

**Authors:** M. Fahad Najam, Nusrat Jafri

**Affiliations:** 1 Anaesthesiology, Abbasi Shaheed Hospital, Karachi, PAK

**Keywords:** acute pain, : pain, pain on vas, less pain, pain

## Abstract

Background and objective

In light of the scarcity of data and research about the management of pain in a low-resource setting, we conducted this study with a view to assessing the effectiveness of intravenous ketamine in comparison to that of intradermal lidocaine in reducing postoperative pain. Postoperative pain can lead to significant morbidity, longer hospital stay, and the development of chronic pain. Our study was formulated to assess the effectiveness of a ketamine bolus in comparison to intradermal lidocaine at the wound site in terms of decreasing pain scores postoperatively.

Methods

In our study, 99 patients were randomly selected to undergo inguinal hernia repair under spinal anesthesia. After obtaining informed consent from the participants and approval from the hospital ethical committee, the patients were randomly classified into the following three groups: the lidocaine group (group A), the ketamine group (group B), and the control group (group C). The patients in the lidocaine group received 0.6 mL/kg of 0.25% lidocaine (1.5 mg/kg) infiltration at the wound site. The ketamine group was given a 50-mg ketamine bolus at the end of the operation, and the control group did not receive either ketamine or intradermal lidocaine at the wound site. Postoperative pain was recorded using the Visual Analog Scale (VAS) scoring and the results were compared. The time of the first request for analgesia was also recorded.

Results

The pain scores measured via VAS scores were higher in patients who received intradermal lidocaine (group A) at the wound site as compared to group B that received a bolus of 50-mg ketamine (p<0.0001); the control group (group C) had pain scores higher than both groups A and B (p=0.0001).

Conclusion

Based on our findings, administering ketamine bolus can significantly decrease VAS scores and reduce the incidence of chronic post-surgical pain as compared to lidocaine infiltration. Ketamine, an N-methyl-D-aspartate (NMDA) receptor antagonist, provides excellent pain relief and analgesia, which decreases overall pain scores.

## Introduction

In an acute postoperative setting, it is essential to provide maximum pain relief to patients to reduce the length of hospital stay and facilitate rapid ambulation. The research on pain management in low-resource settings is minimal. Therefore, we believe that this study, which aims to compare the use of ketamine, an N-methyl-D-aspartate (NMDA) receptor antagonist, with intradermal wound infiltration with 0.6 mL/kg of 0.25% of lidocaine (1.5 mg/kg) to reduce postoperative pain, is very significant. Surgical procedures to treat inguinal hernias are commonly performed, and they can lead to significant postoperative pain. Acute postoperative pain might result in prolonged bed rest, which can cause other medical issues such as thromboembolism. Inguinal hernias are common in the male population. It causes bulging in the area of the groin, leading to acute pain and a decrease in the quality of life. In some cases, an inguinal hernia may also lead to intestinal obstruction [1-2].

The major complications of inguinal hernia repair are recurrence and acute pain. Using 0.6 mL/kg of 0.25% lidocaine (1.5 mg/kg) solution of anesthesia intradermally at the surgical site can reduce the incidence of acute postoperative pain following the open repair of an inguinal hernia. Using local anesthesia infiltration offers many advantages and its use is recommended in open repair techniques. It is recommended that patients start ambulation as soon as possible after surgery. Studies have revealed an increased incidence of postoperative pain in this patient population. Lidocaine is considered to be the least toxic local anesthetic agent. The low dose of 0.6 mL/kg of 0.25% lidocaine (1.5 mg/kg) is considered safe and effective in producing regional blocks [3-4].

Acute postoperative pain decreases over time but debilitating pain may lead to the aggravation of medical conditions due to prolonged bed rest and increases the length of hospital stay. Poor pain management in an acute setting might lead to the development of persistent pain at the site of surgery. Chronic postoperative inguinal pain is a cause for concern as this affects daily work-life balance and decreases the quality of life. It is defined as pain that lasts for around three months and then decreases over time. It has been suggested that local infiltration of the dermis with lidocaine can decrease the incidence of acute pain as well as the incidence of chronic pain. Intravenous ketamine can also provide pain relief by acting on NMDA receptors. Low-dose ketamine bolus may be useful in reducing opioid consumption and preventing the development of chronic pain [5-6].

Infiltration of a wound with 0.6 mL/kg of 0.25% lidocaine (1.5 mg/kg) has been associated with a decrease in pain at the incision site. Lidocaine applied at the end of surgery involving skin and subcutaneous tissue can decrease postoperative pain. Lidocaine works by blocking voltage-gated sodium channels, leading to a decrease in nerve stimulation and transmission of impulses. Patients are regularly operated on for inguinal hernia repair as it is one of the most common surgeries performed in hospitals [7].

## Materials and methods

This study was carried out at the Abbasi Shaheed Hospital in Karachi, Pakistan. After obtaining approval from the hospital ethical committee, we designed a randomized and double-blinded study. The sample size for each group was calculated based on the random sampling method, and it was decided that each group would have 33 participants. The inclusion criteria were men aged 55-75 years without any comorbidities. Those with any systemic disease or any history of previous surgery or use of psychiatric drugs were excluded from the study.

In this comparative trial, 99 patients were randomly selected; all of them were scheduled to undergo inguinal hernia repair under spinal anesthesia. All patients were aged between 55-75 years and had no prior history of abdominal surgery. They were divided into three groups with 33 participants each according to the principles of a randomized sampling method.

Informed consent was obtained from all patients selected for the study. The Visual Analog Scale (VAS) scoring method was clarified to the patients: 0 stands for no pain and 10 represents the worst pain.

Patients scheduled for surgery were randomly selected and divided into the following three groups: the lidocaine group (group A), the ketamine group (group B), and the control group (group C). In the lidocaine group, where all patients were scheduled for unilateral inguinal hernia repair under spinal anesthesia, 0.6 mL/kg of 0.25% lidocaine (1.5 mg/kg) was injected at the wound site when the surgeon completed the operation. In the ketamine group, which also included patients undergoing unilateral hernia under spinal anesthesia, they were given ketamine 50 mg bolus after the completion of the surgery. The control group consisted of patients having unilateral hernia repair under spinal anesthesia who did not receive any intervention for pain management. Postoperative pain was recorded using a VAS scoring system with scores ranging from 1 to 10.

Postoperative pain was evaluated at the post-anesthesia care unit at 30, 60, and 120 minutes postoperatively. The data were analyzed using SPSS Statistics version 26.0 (IBM, Armonk, NY). All quantitative variables such as age, weight, duration of surgery, baseline heart rate, baseline mean arterial pressure, and VAS scores were assessed for their distribution. Age was presented with mean ±standard deviation (SD) while the median (interquartile range) was used for reporting other non-normally distributed variables. The analysis of variances (ANOVA) test was applied to assess the difference between the three groups regarding age. Other statistical differences were identified using the Kruskal-Wallis test. A p-value of ˂0.05 was considered statistically significant. For significant differences, pair-wise comparisons were done and presented with a subscript (as mentioned in Table 2).

## Results

A total of 99 patients were enrolled in this study, 33 in each of the three groups. Table 1 shows the baseline characteristics along with the VAS score at different time points after the surgery. The mean age of patients who were operated on using bupivacaine was 44.2 ±1.1, and it was not statistically different (p=0.985) from the other two groups. Interestingly, the median (IQR) in terms of weight and the duration of surgery were also statistically similar in both groups, with p-values of 0.0973 and 0.856 respectively. Baseline heart rate and mean arterial pressure were also measured for all groups. However, none of these showed a statistically significant difference.

Regarding the VAS score, it was measured at 30, 60, and 120 minutes post-surgery. Table 2 shows the differences between the VAS scores at each time point. At 30 minutes, the median (IQR) VAS score was significantly lower, i.e., 2.0 (2.0-3.0), for the ketamine group compared to the control and lidocaine groups (p<0.0001). However, at 60 minutes, the VAS score became significantly higher for the lidocaine group with a median of 4.0 and a p-value of <0.0001.

**Table 1:** Comparison of baseline characteristics among the three groups *Mean ±standard deviation. ^Median (interquartile range) MAP: mean arterial pressure

Variables	Lidocaine group (n=33)	Ketamine group (n=33)	Control group (n=33)	P-value
Age*, years	44.2 ±1.1	44.5 ±1.1	44.4 ±1.1	0.985
Weight^, kg	67.0 (60.0-78.0)	68.0 (60.0-77.5)	68.0 (60.0-79.0)	0.973
Duration of surgery^, minutes	55.0 (55.0-56.5)	55.0 (55.0-56.0)	55.0 (55.0-56.0)	0.856
Baseline heart rate^, bpm	72.0 (69.5-75.0)	70.0 (69.0-72.0)	72.0 (69.5-75.0)	0.12
Baseline MAP^, mmHg	101.0 (100.0-103.0)	101.0 (99.5-103.0)	100.0 (99.0-102.0)	0.181

**Table 2:** Postoperative comparison of VAS scores among the three groups ^Median (interquartile range). a, b: subscript for pair-wise comparison VAS: Visual Analog Scale

Postoperative time points	Lidocaine group (n=33)	Ketamine group (n=33)	Control group (n=33)	P-value
30 minutes^	5.0 (4.0-6.0)a	2.0 (2.0-3.0)b	5.0 (4.0-5.0)a	<0.0001
60 minutes^	4.0b	3.0 (2.0-3.0)a	3.0 (2.0-3.0)a	<0.0001
120 minutes^	3.0 (3.0-4.0)a	2.0 (1.0-3.0)b	3.0a	<0.0001

## Discussion

Our study aimed to determine whether using a 50-mg bolus of ketamine at the end of inguinal hernia repair was effective in reducing pain in the acute postoperative period. A comparison was made with the use of intradermal 0.6 mL/kg of 0.25% lidocaine (1.5 mg/kg) at the wound site. In low-resource settings, it is critical to use the minimum quantity of drugs available to provide maximum pain relief to patients. Pain management strategies are lacking in terms of guiding toward the best modes of pain relief in low-resource settings or places where there is a scarcity of medicine. It is vital to reduce acute postoperative pain as it might lead to problems such as thromboembolism, increased pressure on hospital resources, and decreased patient satisfaction [8-10].

The lidocaine Infiltration of the surgical wound has been used to sensitize the nerve ending, leading to the decreased entry of noxious and paralyzed stimuli. The study also stresses that infiltration reduces the pain scores in the acute postoperative period. Combination with spinal anesthesia can also contribute to reducing VAS scores and decreasing overall opioid requirements. The baseline values of mean arterial pressure, age, etc., are all presented in Table 1.

In our study, a decrease in VAS scores was seen in the ketamine group, in which a bolus of 50 mg of ketamine was given at the end of inguinal hernia repair. In comparison, the VAS showed a higher score in the lidocaine and control groups. In the control group, at the end of the surgery, no ketamine or intravenous or surgical infiltration was performed. Patients who requested analgesia were given oral non-steroidal anti-inflammatory drugs (NSAIDs) if they experienced pain [11-13].

Table 2 demonstrates the comparison of pain using VAS at different time points. It was shown that mean VAS scores were significantly lower in the ketamine group, indicating effective pain control during the acute postoperative period. At 60 minutes postoperatively, the median VAS scores in the lidocaine group were higher as compared to the ketamine and control groups, indicating the ineffectiveness of lidocaine in reducing pain in the acute postoperative period. At 120 minutes, the median VAS scores were lower in the ketamine group as compared to the lidocaine and control groups (p<0.0001).

The benefits of ketamine have been demonstrated in a similar study in which an intravenous ketamine bolus was used. The ketamine given at a low dose was found to be effective in reducing acute postoperative pain, and patients reported better pain control and higher satisfaction. It also highlighted that patients were managed with minimal clinical resources, and no special care was required [14-15].

## Conclusions

Ketamine bolus is highly effective in decreasing VAS scores and reducing the incidence of chronic post-surgical pain as compared to lidocaine infiltration. Ketamine, an NMDA receptor antagonist, provides excellent pain relief and analgesia, which decreases overall pain scores. Lidocaine infiltration of surgical wounds did not decrease pain scores in the acute postoperative period. While further research is necessary to quantify the long-term effects of ketamine on pain scores, our study provides valuable insights into the management of pain in low-resource settings.
